# Malaria Screening and Treatment in Hematopoietic Cell Transplant Donors and Sickle Cell Disease Candidates/Recipients: A Case Series Using Malaria Polymerase Chain Reaction Testing and a Literature Review

**DOI:** 10.1111/tid.70139

**Published:** 2025-11-28

**Authors:** Mary M. Czech, Sanchita Das, Emily Limerick, Courtney Fitzhugh, Matthew Hsieh, Jennifer Cuellar‐Rodriguez

**Affiliations:** ^1^ Laboratory of Clinical Immunology and Microbiology National Institute of Allergy and Infectious Diseases National Institutes of Health Bethesda Maryland USA; ^2^ Department of Laboratory Medicine National Institutes of Health Bethesda Maryland USA; ^3^ Cellular and Molecular Therapeutics Branch National Heart, Lung, and Blood Institute National Institutes of Health Bethesda Maryland USA

**Keywords:** hematopoietic cell transplant, malaria, polymerase chain reaction, sickle cell disease

## Abstract

**Background:**

Malaria during hematopoietic stem cell transplant (HCT) poses serious risks. Historically, donors with potential exposure were deferred or treated empirically. Malaria PCR, the most sensitive diagnostic tool, is not routinely used. Patients with sickle cell disease (SCD) and their related donors may be disproportionately affected given endemic exposures and potential occult parasitemia.

**Methods:**

Performed a single‐center retrospective review of malaria screening and outcomes in patients with SCD undergoing allogeneic HCT and their related donors. In addition, reviewed the literature on HCT‐related malaria cases.

**Results:**

Among 57 HCT donors tested for malaria, three asymptomatic cases were identified. Two were identified prior to donation via blood smears and PCRs, while one—initially screened with smears alone—was diagnosed retrospectively after transmitting malaria to the recipient. Retrospective malaria PCR of the hematopoietic cell product was positive, suggesting the donor's pre‐collection whole‐blood malaria PCR may have been positive. Among 52 HCT recipients tested for malaria, two developed peri‐HCT malaria—one diagnosed and treated pre‐HCT, and another with donor‐derived malaria. All cases diagnosed before collection and HCT proceeded successfully after treatment and negative PCR. Literature review identified 10 detailed malaria cases in HCT and two additional series lacking case specifics.

**Discussion:**

Asymptomatic HCT donors and candidates with potential exposure to malaria should undergo screening. Malaria PCR offers greater diagnostic sensitivity than conventional methods. PCR utilization may prevent unnecessary donor deferrals and avoided empiric malaria treatment. Moreover, PCR negativity post‐treatment may help confirm donor and candidate eligibility. These observations warrant validation in larger studies.

## Graphical abstract

Malaria polymerase chain reaction (PCR) testing is more sensitive than conventional diagnostics. Routine PCR screening of at‐risk asymptomatic hematopoietic cell donors and transplant candidates may prevent unnecessary deferrals or treatment. In our experience, PCR negativity during screening or after treatment supports donor/candidate eligibility.

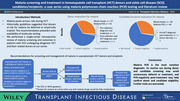

**Abbreviations**
CDC: Centers for Disease Control and Prevention
FDA: Food and Drug Administration
HCT: hematopoietic cell transplant
IQRinterquartile range: interquartile range
PCR: polymerase chain reaction
SCD: sickle cell disease

## 1 Introduction

Malaria after hematopoietic cell transplant (HCT) is an uncommonly reported event, despite malaria being the most common parasitic disease worldwide. This likely reflects the limited number of transplants performed in malaria‐endemic regions, and the low prevalence of at‐risk patients in high‐volume transplant centers located in non‐endemic regions. To date, malaria in the setting of HCT has been reported only in single‐patient case reports and limited case series, the totality of which are summarized in Table 1. In HCT recipients, malaria may arise through donor‐derived transmission (transfusion or hematopoietic cell associated), relapse of a previous infection, or new infection acquired in endemic areas or following travel from such areas [1, 2, 3, 4, 5, 6, 7, 8, 9, 10, 11, 12, 13, 14].

**TABLE 1:** Published cases of malaria in the context of HCT^a^.

Ref	Recipient age/ sex / underlying condition/HCT type	Recipient's malaria risk factors	Recipient screening	Donor's malaria risk factors	Donor screening	*Plasmodium* species infecting recipient/parasitemia	Days from HCT to malaria diagnosis	Recipient treatment	Recipient outcome
**Suspected HCT donor‐derived malaria**									
[1]	14 yo/F/CML/MUD	None Lived in UK, and never traveled outside UK	None reported.	*P. vivax* infection following vacation to Papua New Guinea. Treated and asymptomatic for 11 months pre‐collection.	None reported.	*P. vivax* 1% parasitemia	Day +40	Chloroquine × 7 days, primaquine × 3 weeks	No malaria complication
[2]	27 yo/F/SAA/MRD	From Nigeria, living in UK × 3 years. Traveled to Nigeria 5 months prior to HCT, during which time she was given daily proguanil ppx, and then 200 mg base × 10 tablets on return to the UK.	Negative blood smears pre‐HCT.	From Nigeria. He did not receive any anti‐malaria therapy.	Pre‐HCT blood smears negative before first and second collection. Suspected donor‐derived bc not in an endemic area, recipient was treated pre‐HCT, and donor was suspected to have partial immunity w/ incomplete ability to sterilize malaria.	*P. falciparum* No parasitemia reported.	Day +35 following first HCT, and Day +6 following second HCT	Death before therapy started.	Secondary graft failure, re‐transplanted with same donor, and then death.
[3]	47 yo/M/AML/MRD	From Madagascar, living in France × 25 years. No travel to malaria‐endemic country × 1 year pre‐HCT. Pre‐HCT anti‐*Plasmodium* antibodies negative.	None reported.	From Madagascar, and left residence 2 months pre‐ collection.	No report of donor screening pre‐collection. On Day +18, donor buffy coat test was negative, but positive anti‐*Plasmodium* antibodies. Blood donors for RBCs and platelets in 8 months preceding HCT denied travel to malaria‐endemic countries.	*P. falciparum* 11 840 parasites/µL	Day +15	Quinine + doxycycline × 7 days	No malaria complication.
[4]	30 yo/M/AML/MRD	From Comoro Islands, living in Frace × 8 years. Last travel to malaria‐endemic area > 1 year pre‐HCT. Seropositive for *P. falciparum* and had h/o multiple malaria infections.	Negative blood smears pre‐HCT.	Donor from Comoro Islands, left residence 2 months pre‐collection. Seropositive for *P. falciparum* and had h/o multiple malaria infections.	Negative blood smears pre‐HCT. Suspected to be donor‐derived bc recipient left endemic area > 1 year pre‐HCT.	*P. falciparum* 12.5% parasitemia	Day +12	Quinine × 3 days + halofantrine	No malaria complication
**Suspected transfusion‐transmitted malaria to HCT recipients**									
[5]	25 yo/M/MDS/MRD	None From Tunisia; no autochtonous malaria transmission in Tunisia.	Negative blood smears pre‐HCT.	Donor of infected blood units was a native of a malaria‐endemic area in Africa.	HCT donor blood smears negative pre‐HCT. Retrospective screening of blood unit donor showed infection was acquired from blood units given on Day −5.	*P. falciparum* 23% parasitemia	Day +22	Quinine × 10 days	Malaria reported a/w HLH, but treated with resolution
[6]	27 yo/M/SAA/MRD	None From Tunisia, and no malaria transmission in Tunisia.	None reported.	No risk factors in HCT donor. RBC donor was native of a malaria‐endemic country.	Following recipient diagnosis, RBC donor malaria PCR positive for *P. falciparum*. Thick and thin smears and PCR for malaria in HCT donor were retrospectively negative.	*P. falciparum* 20% parasitemia	Day +26	Quinine × 7 days	Malaria reported a/w HLH, but treated with resolution
**Suspected relapsed malaria**									
[7]	20 yo/M/CML/MRD	From India. Reported h/o malaria.	None reported. Authors report suspicion of pt having relapsed infection.	Presumed from India.	None reported.	*P. vivax* No parasitemia reported.	Day +70	Chloroquine × 3 days, primaquine × 14 days	No malaria complication
[8]	20 yo/F/AML/Auto	From Bangladesh. Did not receive malaria ppx. Last exposure to malaria‐endemic area 7 months pre‐HCT.	None reported.	NA	NA	*P. vivax* 4% parasitemia	Day +14	Chloroquine × 3 days + primaquine × 14 days	No malaria complication
**Malaria source unclear—newly acquired malaria in endemic area vs. donor‐derived vs. relapsed malaria**									
[9]	12 yo/F/beta‐thalassemia major/MRD	From Vietnam.	None reported.	Presumed from Vietnam.	None reported pre‐HCT. Reported that screening of HCT and blood donors did not give positive malaria results post‐HCT (unclear duration of follow up).	*P. falciparum* 2.8% parasitemia	Day +11	Quinine × 7 days + mefloquine × 1 dose	Graft failure w/ autologous reconstitution
[10]	Report six cases of malaria in Brazil in donors (*n* = 2) or recipients (*n* = 4) (out of 953 HCT donor and recipients) that were all identified before HCT. Clinical details regarding proximity to HCT, follow‐up diagnostics, and treatment are not specified.								
[11]	Report that malaria, and specifically transfusion‐transmitted malaria, is a problem during the early transplant period. Further details regarding malaria are not provided.								
**Malaria diagnosed in donor post‐HCT**									
[12]	6 yo/M/ALL/MRD	From the Amazon rain forest.	Blood smear monitoring post‐HCT.	From the Amazon rain forest. Reported nine lifetime episodes of malaria, last *P. vivax* 41 days pre‐HCT.	Treated before collection for *P. vivax*, and then reported post‐HCT monitoring. Donor had asymptomatic positive blood smears for *P. vivax* on Day +27. Retrospective malaria PCR was positive 1 week earlier in the donor.	No documented malaria transmitted to HCT recipient.	Donor diagnosed on Day +26.	Recipient received ppx chloroquine × 3 days d/t possible graft transmitted malaria.	Recipient did not develop malaria through Day +74.

*Note*: All recipients diagnosed by blood smears, and reference [3] additionally diagnosed by buffy coat test.

Abbreviations: ALL—acute lymphoblastic leukemia; Auto—autologous; CML—chronic myeloid leukemia; F—female; HCT—hematopoietic cell transplant; M—male; MDS—myelodysplastic syndromes; MRD—matched related donor; MUD—matched unrelated donor; SAA—severe aplastic anemia; a/w ‐ associated with.

^a^ Excludes three cases of malaria that were previously published from our center, are included in the data of this manuscript, and are further detailed in Table 2.

Malaria poses substantial risk for morbidity and mortality in HCT recipients. Asymptomatic infection may go unrecognized pre‐HCT. Persons from malaria‐endemic countries, who often have partial immunity to malaria from recurring infections, may have pauci‐symptomatic infection, leading to occult and prolonged parasitemia that may persist for years [15]. Furthermore, malaria symptoms, such as fever, anemia, and thrombocytopenia, are non‐specific and may overlap with other peri‐transplant complications, potentially delaying diagnosis and increasing the risk of severe disease.

Prevention of peri‐HCT malaria requires careful screening of potential donors and recipients. To prevent donor transmitted malaria, all donors should be screened by a thorough history to identify potential malaria exposures. Given the paucity of published experience with malaria in the setting of HCT, guidelines for preventing infectious complications among HCT recipients, based on expert consensus, recommend deferring donation for 1 year following travel to malaria‐endemic areas, and for at least 3 years in individuals with prior residence in such areas or a history of malaria [16, 17]. If deferral is not feasible, or if donors remain in malaria‐endemic regions, empiric malaria treatment is recommended [10, 16, 17, 18]. Historically, malaria testing alone is not recommended, particularly in donors from endemic regions, given concerns for false‐negative results and occult low‐level parasitemia that may evade microscopic and antigenic detection. However, this approach may result in unnecessary exclusion or treatment of otherwise eligible donors [18]. It is important to note that these guidelines addressing malaria prevention peri‐HCT predate the broader implementation of malaria molecular testing. This situation is akin to the historic unnecessary exclusion of transplant donors due to fears of transmitting hepatitis C virus, hepatitis B virus, or human immunodeficiency virus, prior to the routine implementation of molecular testing, which ultimately enhanced donor utilization [19].

For HCT candidates from malaria‐endemic areas, pre‐transplant screening is recommended, and post‐transplant diagnostics are recommended if there is a clinical syndrome compatible with malaria [16, 17]. Candidate/recipient screening may include blood smears, rapid antigen tests, and/or polymerase chain reaction (PCR) testing [16].

Malaria PCR testing is the most sensitive assay [20, 21, 22]. Historically, PCR has not been included in the diagnostic algorithm due to its relatively slow turnaround time compared to the more immediate results from direct microscopy and rapid tests, and the limited availability of PCR in resource‐constrained settings. However, these limitations are less significant for asymptomatic HCT donors/candidates, and HCT centers already use molecular diagnostics platforms for routine peri‐transplant care.

Sickle cell disease (SCD) is an inherited hemoglobinopathy that results in significant disability and mortality worldwide. Allogeneic HCT is a curative intervention for SCD. Outside of North America, SCD disproportionately affects patients in malaria‐endemic regions [23, 24]. Patients with SCD are more vulnerable to severe and complicated malaria [25]. Sickle cell trait, common in related HCT donors, is protective against severe and complicated malaria which may result in asymptomatic or minimally symptomatic infection [25]. Therefore, careful malaria screening is essential for both donors and candidates at‐risk for malaria. Access to HCT for SCD remains limited. However, our group has been developing HCT strategies for domestic and international SCD patients for many years.

This report outlines our experience using malaria PCR to screen asymptomatic donors and HCT candidates, and to guide the management of identified infections. In addition, we review the current published experience surrounding malaria and HCT.

## 2 Methods

This study is a single center, retrospective chart review of malaria and screening for malaria in patients with SCD who received allogeneic HCT and their related donors between July 2004 and February 2024. All donors and recipients gave written consent to participate in National Institutes of Health Institutional Review Board‐approved protocols. Our center, located in the United States of America, is not located in a malaria‐endemic region. However, a significant proportion of our patients either live in or travel to international areas where malaria is prevalent.

All patients with SCD who underwent HCT were included. All HCT donors to SDC patients who were collected were included. No individuals were excluded from analysis. Patient charts were reviewed to detail information regarding malaria diagnostics and treatments.

Based on our own institutional guidelines for the prevention of infections in HCT recipients, HCT donors and candidates are recommended for malaria evaluation if they resided in or traveled to areas endemic for malaria (list of malaria‐endemic countries updated by the Centers for Disease Control and Prevention [CDC] [26]). The timeframe during which residence or travel to a malaria‐endemic area was considered a risk for malaria was determined by the physician's assessment of the duration and intensity of exposure in an endemic area, likelihood of malaria partial immunity, and local prevalence of *Plasmodium* species (*Plasmodium vivax*, *Plasmodium ovale*, and *Plasmodiummalariae* known to cause subclinical or relapsing infection). Due to incomplete documentation of residence and travel history to malaria‐endemic regions in medical records, donors and recipients tested for malaria were analyzed irrespective of confirmed exposure risk. In addition, recipients tested for malaria within 100 days following HCT, which is a period during which they would remain local to our center, in an area not endemic for malaria, were also analyzed.

Historically, the recommendation that HCT donors at‐risk for malaria either defer donation or receive empiric malaria treatment [10, 16, 17, 18] has been based on expert opinion. Therefore, there are no standardized microbiologic screening guidelines for asymptomatic HCT donors at‐risk of malaria, and nor are there established testing protocols for candidates/recipients. Asymptomatic malaria evaluation at our institution has evolved over time. A minority of donors and recipients were prescribed empiric malaria treatment or prophylaxis based on provider discretion. Most malaria screening employed a combination of microscopy to evaluate thick and thin blood smears, and PCR testing. Malaria PCR has been available at our center starting in 2011 (6 years into the study period). The individual number of blood smears and PCR tests collected per patient was not standardized. Rapid antigen tests for malaria were not available at our center during the study period; although these assays can aid in the diagnosis of symptomatic malaria, their sensitivity is lower compared to PCR [20].

### 2.1 Molecular Testing

Malaria PCR testing was conducted on whole blood using a laboratory‐developed assay. The multiplexed real‐time PCR assay is performed using Taqman probes targeted to the 18S ribosomal subunit. There are five targets, a genus specific malaria target (157 bp long) and four species specific targets for *Plasmodium falciparum, P. vivax, P. ovale, and P. malariae* (ranging in size from 104‐112 bp). The limit of detection is approximately 0.6 parasites of *Plasmodium*/µL of blood.

## 3 Results

### 3.1 HCT Donors

There were 157 collected donors during the study period. Of these, 56 were tested for malaria before collection, and one donor who was evaluated for malaria 4 days following the collection date was also analyzed. Among these 57 donors evaluated for malaria, 39 had sickle cell trait and three had beta‐thalassemia trait.

Travel history to malaria‐endemic areas was incompletely documented, but countries of birth for donors evaluated for malaria included: Nigeria (*n* = 19), USA (*n* = 4), South Africa (*n* = 3), Caribbean (*n* = 2), Kenya (*n* = 2), Uganda (*n* = 2), Benin (*n* = 1), Cuba (*n* = 1), Democratic Republic of the Congo (*n* = 1), Ghana (*n* = 1), Honduras (*n* = 1), Pakistan (*n* = 1), Sierra Leone (*n* = 1), The Gambia (*n* = 1), Tunisia (*n* = 1), United Kingdom (*n* = 1), and unknown (*n* = 15).

Among the 57 donors evaluated for malaria, all had blood smears and most had PCR testing. Among 27 total donors collected before malaria PCR was available at our center, four (15%) of these patients were evaluated for malaria with blood smears alone. Following the availability of malaria PCR, a total of 131 donors were collected, of which 53 (40%) were evaluated with blood smears and concurrent PCR. The median number of smears for malaria donor evaluation was 1.0 (interquartile range, IQR 0.0, range 1–5). The median number of PCRs for malaria donor evaluation was 1.0 (IQR 0.0, range 0–5). The first malaria evaluation was at a median of 20 days (IQR: 13.8–25.0) before stem cell collection.

Seven donors tested for malaria received empiric treatment for malaria preceding the donation date. This practice was in keeping with historic guidelines recommending empiric treatment (or deferral) for at‐risk donors [16, 17]. Four donors were tested for malaria before receiving empiric treatment, whereas three donors were tested after receiving empiric treatment; it is not clear from chart review why empiric treatment was initiated before malaria testing for these three donors. All seven had malaria blood smears performed and five underwent PCR testing; PCR testing was not performed for two patients that were evaluated before malaria PCR testing was available at our center. Empiric malaria treatment regimens included atovaquone/proguanil (*n* = 5), doxycycline (*n* = 1), and mefloquine (*n* = 1). None of these seven empirically treated patients were found to have malaria at any time point.

Three donors were diagnosed with malaria peri‐collection date (Table 2). All donors with malaria originated from malaria‐endemic regions and were asymptomatic at the time of initial evaluation. Two donors were diagnosed and treated for malaria preceding the donation date, while another donor was diagnosed with malaria only after transmission to the HCT recipient was identified.

**TABLE 2:** Cases of malaria in HCT donor and recipients at our center.

Donor or recipient with malaria	Age/sex/underlying condition	Place of origin	Malaria testing	Infecting *Plasmodium* species/ parasitemia	Days from malaria diagnosis to/ from collection or HCT	Treatment	Outcome
Donor	28 yo/F/sickle cell trait	Uganda	Positive smear and PCR pre‐collection	*P. ovale* No quantifiable parasitemia –seen in thick smear only	28 days pre‐collection	Hydroxychloroquine × 4 doses + primaquine × 14 days	No malaria complication
Donor	30 yo/F/sickle cell trait	Nigeria	Positive smear and PCR pre‐collection	*P. falciparum* No quantifiable parasitemia –seen in thick smear only	9 days pre‐collection	Atovaquone‐proguanil × 3 days	No malaria complication
Donor [13]	16 yo/M/sickle cell trait	Sierra Leone	Smear negative × 3 pre‐collection. Retrospective pre‐collection plasma antigen positive, and PCR of HCT product positive.	*P. falciparum* No quantifiable parasitemia (only antigen and PCR positive)	94 days post‐collection (61 days post‐collection to recipient malaria diagnosis)	Atovaquone‐proguanil × 3 days	Occult malaria undiagnosed pre‐collection. Transmitted malaria to HCT recipient
Recipient [13]	25 yo/F/HbSS	Sierra Leone	No pre‐HCT malaria evaluation. Post‐HCT test positive.	*P. falciparum* 5.2% parasitemia	Day +11 post‐HCT—donor‐derived infection	Atovaquone‐proguanil × 6 days	Secondary graft failure. Re‐transplanted with same donor, and again had graft failure.
Recipient [14]	32 yo/M/HbSS	Nigeria	Initial smear and PCR negative (2 weeks following empiric artemether/lumefantrine × 3 days). Repeat smear and PCR positive 19 days following initial test.	*P. falciparum* 2.9% parasitemia	Day −41 pre‐HCT	Artemether‐lumefantrine × 3 days, atovaquone‐proguanil × 7 days, doxycycline × 3 days	No malaria complication

The two donors found to have malaria before the collection date were diagnosed by positive blood smear and malaria PCR.

One donor, a 28‐year‐old female with sickle cell trait, was diagnosed with *P. ovale* 28 days prior to donation by blood smear and PCR. She traveled from her residence in Uganda to our center 2 days prior to her positive malaria test. She was initially asymptomatic, but subsequently developed fevers. She was treated with hydroxychloroquine for four doses and primaquine for 14 days. A single follow‐up malaria PCR was negative 20 days following the initial positive test, coinciding with the day treatment was completed. No further follow‐up testing was conducted. Collection occurred 9 days following the completion of malaria treatment, and the subsequent transplant proceeded without malaria complications.

The other donor, a 30‐year‐old female with sickle cell trait, was diagnosed with *P. falciparum* 9 days prior to donation by blood smear and PCR. This donor arrived from her residence in Nigeria 3 days prior to her positive malaria test. She was asymptomatic, and notably had a history of malaria with the last infection occurring a year prior. She was treated with atovaquone‐proguanil for 3 days and had a negative smear and PCR by 2 days following the completion of treatment. Four days following the completion of treatment, on the day of donation, a repeat smear and PCR remained negative. Donation was unremarkable with no recrudescence of infection or identification of malaria in the HCT recipient.

The donor who transmitted malaria to the HCT recipient was a 16‐year‐old male with sickle cell trait. He was only evaluated with blood smears, not PCR, prior to donation. This case has been previously described [13]. The donor, from Sierra Leone, was asymptomatic with two malaria blood smears negative 45‐ and 41‐days preceding peripheral blood hematopoietic cell collection. A subsequent third malaria smear was negative post‐collection and pre‐HCT. Malaria PCR testing was not clinically available at our center at that time, so it was not performed prior to donation. Following the HCT recipient's malaria diagnosis on Day +11 post‐HCT, the donor was tested and found to have *P. falciparum*. Retrospective evaluation of donor whole blood pre‐donation was not available, but stored donor plasma was sent out to a referral laboratory for antigen evaluation and found to be positive. Furthermore, retrospective malaria PCR of the hematopoietic cell product was positive, and genotyping confirmed shared markers between donor and recipient *P. falciparum* strains.

### 3.2 HCT Recipients

During the study period, 152 patients were recipients of allogeneic HCT. A total of 52 patients were tested for malaria—45 patients before HCT, and an additional seven patients with fever within the first 100 days following HCT. Among these 52 patients tested for malaria, SCD genotypes included sickle cell anemia (HbSS) (*n* = 47) and sickle beta‐zero thalassemia (*n* = 3), and two additional patients had beta thalassemia major.

Travel history to malaria‐endemic areas was incompletely documented, but countries of birth for these patients were: Nigeria (*n* = 17), USA (*n* = 11), Democratic Republic of the Congo (*n* = 3), Ghana (*n* = 3), Kenya (*n* = 2), Sierra Leone (*n* = 2), United Kingdom (*n* = 2), Cameroon (*n* = 1), Colombia (*n* = 1), Cuba (*n* = 1), France (*n* = 1), The Gambia (*n* = 1), Honduras (*n* = 1), Iran (*n* = 1), Pakistan (*n* = 1), Trinidad (*n* = 1), Tunisia (*n* = 1), and Uganda (*n* = 2).

Among the 45 recipients evaluated for malaria, all had blood smears and most had PCR testing. Among 24 total recipients transplanted before malaria PCR was available at our center, one (4%) patient was evaluated for malaria with blood smears alone. Following the availability of malaria PCR, a total of 128 recipients were transplanted, of which 44 (34%) were evaluated with blood smears and concurrent PCR. The median number of smears for malaria recipient evaluation was 1.0 (IQR 1.0, range 1–3). The median number of PCRs for malaria recipient evaluation was 1.0 (IQR 1.0, range 0–3). The first malaria evaluation was a median of 65 days (IQR: 38–92 days) preceding transplant. For the 14 patients with more than one blood smear and/or PCR to evaluate for malaria, the evaluation most proximal to pre‐HCT occurred a median of 39 (IQR: 22–57 days) days before HCT.

Only one patient received prophylactic malaria treatment preceding HCT. This patient was prescribed mefloquine while still residing in Nigeria, and it was continued for 5 weeks following arrival at our center. This patient did not develop malaria.

Only one patient had malaria diagnosed before HCT (Table 2). This patient case was previously described as a notable instance of prolonged malaria PCR positivity in the setting of functional asplenia due to SCD [14]. In brief, a 32‐year‐old male with HbSS, received an empiric course of artemether/lumefantrine for 3 days while asymptomatic in Nigeria. Two weeks later, while still asymptomatic, an initial malaria blood smear and PCR were negative at our center. Nineteen days following the initial malaria evaluation a repeat smear and PCR were positive for *P. falciparum* during a fever evaluation. Retrospective evaluation of the initial blood sample using a research‐based, highly sensitive *Plasmodium* 18S rRNA qRT‐PCR assay with a lower limit of detection was positive. Due to prolonged parasitemia and then PCR positivity, the patient received an extended treatment course. The patient ultimately cleared the blood smears after 10 days, and the malaria PCR returned negative 31 days following the start of treatment. After a single negative PCR, the patient underwent an uneventful HCT 10 days later with no recurrence of malaria parasitemia.

Another patient, a 25‐year‐old female with HbSS, was diagnosed with malaria 11 days following peripheral blood HCT in the setting of neutropenic fevers (Table 2). As discussed above, this case was previously described [13] as a case of donor‐derived malaria due to *P. falciparum*.

Otherwise, no other cases of malaria were diagnosed within 100 days post‐HCT. The only other documented case of post‐HCT malaria at our center occurred approximately 3.5 years after HCT as a newly acquired infection in a patient who resumed residence in Uganda and was diagnosed with malaria during a follow up visit at our center.

## 4 Discussion

This is the first case series to describe the routine use of malaria PCR testing in HCT candidates/recipients and their related donors. Our retrospective review identified three cases of malaria peri‐donation in initially asymptomatic HCT donors. Two donors were diagnosed and treated for malaria preceding the donation date. One donor was screened only with blood smears and was unfortunately diagnosed with malaria only after transmission to the HCT recipient was identified. In addition, we identified one case of malaria in an HCT candidate, who was treated and successfully transplanted, and one in an HCT recipient which was donor‐derived.

Aside from our center's data, ten individual case reports of malaria in the context of HCT have been published [1, 2, 3, 4, 5, 6, 7, 8, 9, 12] (summarized in Table 1). One additional study from Brazil reports two donors and four recipients with malaria preceding HCT, but details regarding infection proximity to HCT, follow‐up diagnostics, and malaria outcomes are not reported [10]. Another report from Pakistan noted transfusion‐transmitted malaria as an issue during the early post‐HCT period, though further information was not provided [11]. Among the individual case reports, four cases were suspected HCT donor‐derived malaria, two were transfusion‐transmitted malaria to HCT recipients, two were relapsed infections, one had an unclear source, and one involved malaria diagnosed in a donor post‐HCT. Among seven recipients at‐risk for malaria pre‐HCT, only two patients had pre‐HCT screening with blood smears [2, 4]. Similarly, among eight at‐risk HCT donors, only three had pre‐HCT screening with blood smears [2, 4, 5]. Notably, no cases report pre‐HCT use of malaria PCR for screening donors or HCT candidates, primarily because most cases were reported before malaria PCR became available. In one report, retrospective malaria PCR was positive for an RBC donor and negative for the HCT donor [6]. Another report described a case where retrospective PCR testing was positive for the HCT donor 1 week prior to the detection of malaria on blood smears, which became positive on Day +27 following transplant [12]. *P. falciparum* was the most frequently identified infection (*n* = 6), followed by *P. vivax* (*n* = 4). Donor‐derived malaria occurred a median of +25 days post‐HCT (range +12 to +40 days), while relapsed cases occurred at Days +14 and +70 following HCT. Only one malaria‐related death was reported [2].

For HCT donors at‐risk for malaria, prior guidelines—established before widely available molecular diagnostics—recommended donor deferral or empiric treatment rather than screening given concerns for false‐negative testing with microscopy and antigen tests [10, 16, 17, 18]. For non‐HCT blood product donation, most regions recommend deferral in non‐endemic areas [27, 28], and the World Health Organization (WHO) recommends screening with blood smears and antigen testing in malaria endemic areas [29]. However, malaria molecular testing has been retrospectively reviewed in cases of non‐HCT transfusion‐transmitted malaria and screening asymptomatic non‐HCT blood donors with significant improved sensitivity compared to traditional diagnostics [27]. Using this data, the U.S. Food and Drug Administration (FDA) recently issued updated draft guidance regarding recommendations to reduce the risk for transfusion‐transmitted malaria in January 2025. Whereas prior guidance from 2022 recommended deferral for at‐risk donors [28], the current draft alternatively recommends testing blood donations from these donors at‐risk for malaria using an FDA‐licensed donor screening nucleic acid test (NAT) for *Plasmodium* species [30]. The Cobas malaria test, manufactured by Roche Molecular Systems (Basel, Switzerland), is the first FDA‐approved molecular test to screen for malaria in blood donors.

In the setting of HCT, malaria molecular testing seems highly sensitive to evaluate asymptomatic donors. Two cases were diagnosed with PCR in conjunction with blood smears. The third case had negative blood smears, highlighting that blood smears alone are likely inadequate to evaluate asymptomatic donors. However, retrospective evaluation of the peripheral blood hematopoietic cell product was malaria PCR positive, suggesting that the donor's pre‐collection whole blood malaria PCR may have been positive. Another report from Brazil additionally cites an instance in which malaria PCR diagnosed *P. vivax* in an asymptomatic HCT donor 1 week earlier than thick blood smears, which facilitated preemptive treatment of the HCT recipient who had already received the product [12].

Malaria PCR is now used as the gold standard in malaria research because it is highly sensitive and able to detect infection negative by microscopy and rapid diagnostic tests [20, 21, 22], and it will likely replace other diagnostic modalities in the screening of blood products as suggested by the FDA [30]. PCR has a theoretical detection of 0.5–5 parasite/µL, compared to microscopy where visual inspection has a sensitivity of 100 parasites/µL, depending on the experience of the microscopist [31, 32]. Therefore, routine use of malaria PCR in screening at‐risk asymptomatic donors may obviate the need for delaying donation and/or giving empiric malaria treatment.

In the case of malaria that occurred in a candidate pre‐HCT, malaria smear and PCR were initially negative approximately 2 weeks after the patient received empiric artemether/lumefantrine for 3 days, but then subsequently positive in the setting of fever. Initial malaria evaluation was potentially falsely negative because of the long half‐life of lumefantrine [33]. This case highlights the importance of identifying the timing of malaria prophylaxis and treatment regimens as these therapies may result in initially false negative malaria evaluations. When donors and candidates have received therapies effective against malaria, malaria evaluation should be repeated when these drugs would have been fully metabolized. Other theoretical reasons for false negative malaria PCR may include very low parasite density, especially in persons with partial immunity to malaria, sample handling and processing errors, and technical errors.

Malaria PCR may also be used in positive donors and pre‐HCT candidates to monitor infection status and help determine the appropriate timing for collection and transplant, respectively. In malaria‐endemic areas, the WHO recommends deferring non‐HCT blood product donors for 6 months after completing treatment and full recovery, whichever is longer [29]. In our study, two donors diagnosed with malaria pre‐collection received treatment and were subsequently collected 9 and 4 days after completing treatment. By the time of collection, both donors tested PCR‐negative. Malaria was not transmitted in either HCT product. The HCT candidate diagnosed with malaria pre‐HCT was treated until PCR‐negative, and then underwent HCT without malaria recurrence. These data suggest that PCR reversion to negative may serve as an indicator of donor and transplant eligibility.

We report a case of neutropenic fever caused by malaria in an HCT recipient. This underscores the importance of including malaria in the differential diagnosis for early post‐transplant fevers in HCT recipients and/or donors at‐risk for malaria.

This review has several limitations. It is a single‐center experience focused on SCD, which may restrict the generalizability of the findings. There is incomplete travel and residence data, which may introduce biases in the number of individuals tested for malaria. The evaluation is retrospective with evolving practice patterns and availability of malaria PCR during the study period. Specifically following the introduction of malaria PCR, a higher proportion of donor and HCT candidates underwent malaria testing compared with the pre‐PCR period, possibly reflecting a change in clinical awareness or testing patterns. In addition, there is a low incidence of malaria in HCT donors and candidates/recipients in our center, which limits definitive conclusions about malaria PCR.

**FIGURE 1:**
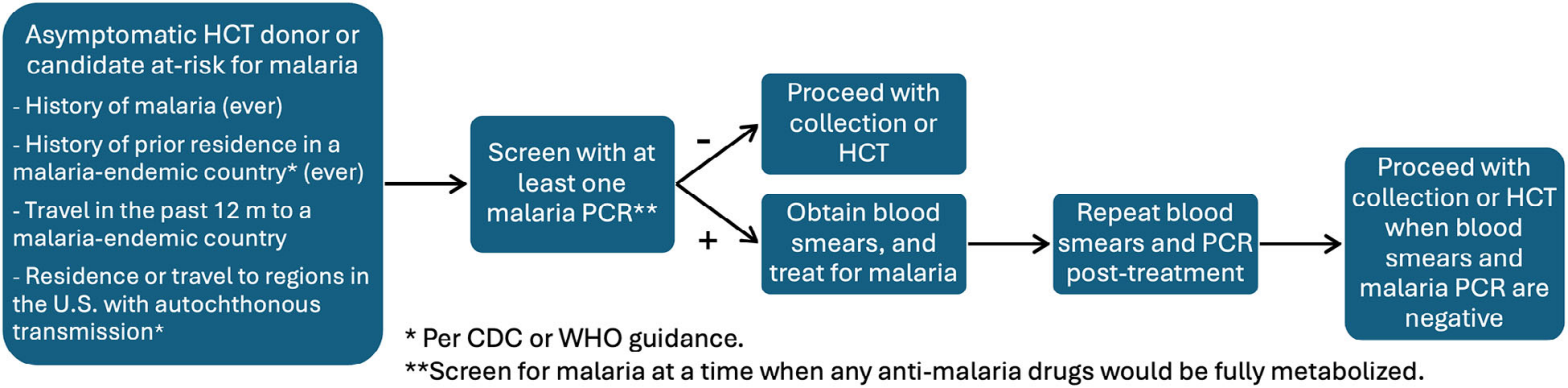
Recommendations for screening and management of malaria in asymptomatic HCT donors and recipients.

Despite its limitation, this review constitutes the only published evaluation examining the role of malaria PCR testing in screening at‐risk asymptomatic donors and HCT candidates /recipients and for evaluating readiness for donation or transplantation. More studies are needed to assess the risk of malaria in HCT. However, we believe that malaria PCR, when available, could be included in routine screening of asymptomatic HCT donors and candidates with potential malaria exposure given its higher sensitivity. This approach may help avoid donor deferral, especially when no other donors are available, and avoid unnecessary empiric malaria treatment. Our recommendations for malaria screening and management in at‐risk asymptomatic HCT donors and candidates are outlined in Figure 1. Akin to guidance provided by the FDA for transfusion‐transmitted malaria [30], we consider persons at‐risk for malaria based on the following: history ever of malaria, history of ever prior residence in a malaria‐endemic country, history in the past 12 months of travel to a malaria‐endemic country, and/or residence or travel to regions in the US with autochthonous transmission (per CDC guidance). We recommend conducting at least a one‐time malaria screening for at‐risk asymptomatic HCT donors and candidates using malaria PCR when no active anti‐malaria drug metabolites are present. The benefit of serial PCR for malaria detection remains unproven, but it should be considered in individuals who recently received malaria prophylaxis or treatment. If malaria is diagnosed, appropriate treatment should be administered, followed by repeat blood smear and PCR testing as a test of cure. We suggest collection and HCT be deferred until both smears and PCR return negative. Given the rarity of malaria infection in the HCT population, demonstrating the superiority of molecular screening for at‐risk donors and candidates, and its potential to prevent unnecessary transplantation delays or empiric treatments, will require large multicenter studies.

## Funding

This research was supported by the Division of Intramural Research, National Institute of Allergy and Infectious Diseases, and the National Heart, Lung, and Blood Institute, and the National Institute of Health Clinical Center, all part of the National Institutes of Health.

## Conflicts of Interest

The authors declare no conflicts of interest.
